# Ticagrelor: A Rare, Delayed Case of Angioedema

**DOI:** 10.7759/cureus.38606

**Published:** 2023-05-05

**Authors:** Jasmit Walia, Ammar Alam, Zarian Prenatt, Timothy Daly, Shannon Kearney

**Affiliations:** 1 Internal Medicine, St. Luke's Hospital, Bethlehem, USA; 2 Internal Medicine, St. Luke's University Health Network, Bethlehem, USA; 3 Allergy and Immunology, St. Luke's Hospital, Allentown, USA

**Keywords:** facial angioedema, drug-induced angioedema, drug-induced hypersensitivity, drug eluting stent, drug-related side effects and adverse reactions

## Abstract

Dual antiplatelet therapy (DAPT) with P2Y12 receptor inhibitors in conjunction with aspirin is the gold standard treatment for acute coronary syndrome (ACS) and to prevent stent thrombosis after percutaneous coronary intervention (PCI). While there have been reported allergic effects-particularly angioedema-linked to clopidogrel there is limited data on hypersensitivity reactions to ticagrelor. Here, we discuss a case of delayed-onset ticagrelor-induced angioedema in a patient, three weeks following initiation of DAPT with aspirin and ticagrelor status post-PCI with DES placement. The patient presented with acute onset tongue swelling and was successfully treated with epinephrine, steroids, and antihistamine. The C1 esterase inhibitor and tryptase levels were within normal limits. Ticagrelor was discontinued and the patient was transitioned to prasugrel for DAPT, without recurrence of symptoms. Given the few cases reported involving ticagrelor-induced angioedema, and the even rare, delayed onset cases such as those described above, it is imperative that clinicians be made aware of this adverse effect and its management.

## Introduction

Dual antiplatelet therapy (DAPT) with P2Y12 receptor inhibitors (clopidogrel, prasugrel, or ticagrelor) in conjunction with aspirin is a standard regimen in the management of acute coronary syndrome (ACS) and to prevent stent thrombosis in patients after a percutaneous coronary intervention (PCI) [1]. It has been well-documented that DAPT with clopidogrel reduces the one-year incidence of acute cardiovascular events by about 20%, compared with acetylsalicylic acid (ASA) monotherapy alone [2]. However, the use of prasugrel and ticagrelor is surging due to their more potent P2Y12 inhibition compared to clopidogrel, as evidenced by the TRITON and PLATO trials, respectively [2,3]. Although adverse drug reactions (ADRs) are more common with aspirin, all patients with ACS receiving DAPT should be monitored for hypersensitivity reactions [4]. While there have been reported allergic effects, particularly angioedema-linked to clopidogrel, there is limited clinical data on hypersensitivity reactions to ticagrelor, including a potentially life-threatening delayed-onset angioedema [5]. Angioedema, characterized by localized tissue swelling caused by fluid leakage into the interstitial tissue, can be further classified into bradykinin-mediated (e.g., inherited or acquired C1 inhibitor deficiency, or angiotensin-converting enzyme (ACE) inhibitor-induced) or histamine-mediated (e.g., direct mast cell activation, or IgE-mediated allergic reactions) [6]. Here, we discuss a case of delayed-onset ticagrelor-induced angioedema in a patient who was initiated on DAPT with aspirin and ticagrelor for the treatment of an ST-segment elevation myocardial infarction (STEMI) status post-PCI with drug-eluting stent placement. With the increasing use of ticagrelor as a first-line agent for ACS, it is vital to have such rare occurrences be reported to databases that are readily available to the medical community. 

## Case presentation

Our patient was a 71-year-old female with a past medical history of hypertension, hyperlipidemia, insulin-dependent type 2 diabetes mellitus, and chronic kidney disease stage 3b. She presented with a two-day history of chest pain that radiated to the left arm. On admission, her EKG showed ST elevation in the inferolateral leads and Q waves in the inferior leads. Her initial high-sensitivity troponins were 11,690, 11,025, and 15,531 at zero hours, two hours, and four hours, respectively. She was given aspirin 324 mg, ticagrelor 90 mg, and started on a heparin drip. Subsequent cardiac catheterization revealed one-vessel coronary artery disease with 80% stenosis of the mid-left descending artery (LAD). The patient underwent successful drug-eluting stent placement and was discharged on DAPT with aspirin 81 mg daily and ticagrelor 90 mg daily. Additional new medications at discharge included metoprolol succinate. Notably, the patient had previously taken metoprolol succinate and aspirin in the past without any prior allergic reactions.

Three weeks later, the patient presented to the emergency department with acute onset tongue swelling, difficulty speaking, and voice change. She reported compliance with the home medications that were prescribed during her previous hospital stay. She also reported having salmon for dinner the night prior but had no prior history of an allergic reaction to seafood. In the ED, the patient had a muffled voice but was controlling her secretions. The physical exam revealed an enlarged tongue nearly completely obscuring the posterior oropharynx without respiratory compromise. She received an epinephrine 0.3 mg intramuscular (IM) injection, methylprednisolone 125 mg IV, and diphenhydramine 50 mg IV. Although the patient had minimal improvement in tongue swelling, she was saturating 98% of the room air. She was admitted to critical care for airway monitoring and started on dexamethasone 6 mg IV every six hours to reduce airway swelling. Overnight, the patient had a significant improvement after receiving four additional doses of dexamethasone and was transitioned to prasugrel 10 mg. Relevant workup included levels of C1 esterase inhibitor and tryptase, both of which were within normal limits. The patient tolerated prasugrel without recurrence of symptoms and was discharged on prasugrel 10 mg daily and aspirin 81 mg daily for maintenance of DAPT.

## Discussion

The incidence of drug-induced angioedema is estimated to be less than 1%, and it most commonly occurs in association with ACE inhibitors [7]. According to the U.S. National Library of Medicine (NLM), hypersensitivity reactions to ticagrelor are uncommon, and 34 cases were reported to have an adverse effect with rashes by the U.S. Food and Drug Administration [8]. Ticagrelor-induced angioedema, which has been documented in two out of the 34 reported cases, is even rarer [9]. However, in the cases collected by NLM and additional instances reported throughout the literature, the onset of these reactions is immediate, usually occurring within hours of exposure [7-14]. The most delayed reaction reported in the literature occurred approximately 36 hours after the first dose of ticagrelor [15]. This case reports a rare occurrence of angioedema with delayed onset, approximately 20 days after the initiation of ticagrelor. The mechanism of angioedema is due to bradykinin release or mast cell activation, but there is a time delay contrary to type 1 hypersensitivity reactions, which are IgE-mediated [9]. 

Angioedema is generally self-limited but can be life-threatening with airway involvement that can quickly progress to respiratory compromise and hemodynamic instability. Treatment revolves around discontinuing the medication, and management differs between bradykinin-mediated and histamine-mediated angioedema [14]. Histamine-mediated angioedema is treated with antihistamines, steroids, as well as epinephrine if the airway is involved. On the other hand, bradykinin-mediated angioedema is generally unresponsive to antihistamines and can be treated with tranexamic acid, ecallantide, icatibant, and/or purified C1 inhibitor concentrate [9]. In this case, the patient responded adequately to a combination of steroids, antihistamine, and an initial dose of epinephrine, indicating a histamine-mediated hypersensitivity reaction. Improvement after discontinuing the medication provides further evidence that the adverse reaction was likely triggered by ticagrelor. 

Ticagrelor, prasugrel, and clopidogrel are adenosine-diphosphate (ADP) receptor (P2Y12) blockers. However, they differ in their underlying chemical structures and can thus be further subdivided. Clopidogrel and prasugrel are classified as thienopyridines, whereas ticagrelor is categorized as cyclopentyl-triazole-pyrimidine [15]. Although both clopidogrel and prasugrel were valid alternatives to ticagrelor in our patient, we opted for prasugrel, given its more potent inhibition and decreased incidence of cardiovascular death, myocardial infarction, and stroke [2]. Additionally, previous studies have shown success with the desensitization of clopidogrel in patients presenting with clopidogrel-associated angioedema. However, no such ticagrelor desensitization protocol has been described in the literature [15]. 

Given the uncommon number of cases reported involving ticagrelor-induced angioedema, and the even rarer, delayed onset case such as those described above, it is essential that clinicians are aware of this adverse effect. Both early detection and management modalities should be emphasized, and patients should be made aware of potential symptoms to facilitate timely identification and treatment.

## Conclusions

In summary, we report a case of ticagrelor-induced angioedema that occurred 20 days after drug initiation. The patient responded well to treatment with epinephrine, steroids, and antihistamines, and most importantly, discontinuation of the offending drug. The patient was promptly transitioned to prasugrel, a structurally dissimilar P2Y12 receptor inhibitor, without any interruption in DAPT.
